# A new role for interferon gamma in neural stem/precursor cell dysregulation

**DOI:** 10.1186/1750-1326-6-18

**Published:** 2011-03-03

**Authors:** Janine Walter, Silke D Honsek, Sebastian Illes, Jennifer M Wellen, Hans-Peter Hartung, Christine R Rose, Marcel Dihné

**Affiliations:** 1 Department of Neurology, Heinrich-Heine-University, Moorenstr. 5, 40225 Düsseldorf, Germany; 2 Institute for Neurobiology, Heinrich-Heine-University, Universitätsstr. 1, 40225 Düsseldorf, Germany; 3 Dept. of Neurophysiology, Center for Brain Research, Medical University of Vienna, Spitalgasse 4, 1090 Vienna, Austria

## Abstract

**Background:**

The identification of factors that compromise neurogenesis is aimed at improving stem cell-based approaches in the field of regenerative medicine. Interferon gamma (IFNγ) is a main pro-inflammatory cytokine and up-regulated during several neurological diseases. IFNγ is generally thought to beneficially enhance neurogenesis from fetal or adult neural stem/precursor cells (NSPCs).

**Results:**

We now provide direct evidence to the contrary that IFNγ induces a dysfunctional stage in a substantial portion of NSPC-derived progeny *in vitro *characterized by simultaneous expression of glial fibrillary acid protein (GFAP) and neuronal markers, an abnormal gene expression and a functional phenotype neither typical for neurons nor for mature astrocytes. Dysfunctional development of NSPCs under the influence of IFNγ was finally demonstrated by applying the microelectrode array technology. IFNγ exposure of NSPCs during an initial 7-day proliferation period prevented the subsequent adequate differentiation and formation of functional neuronal networks.

**Conclusions:**

Our results show that immunocytochemical analyses of NSPC-derived progeny are not necessarily indicating the correct cellular phenotype specifically under inflammatory conditions and that simultaneous expression of neuronal and glial markers rather point to cellular dysregulation. We hypothesize that inhibiting the impact of IFNγ on NSPCs during neurological diseases might contribute to effective neurogenesis and regeneration.

## Background

Neural stem/precursor cells (NSPCs) may be useful as an endogenous or transplantable source of newly generated neural cells, which can replace lost or diseased neurons within the central nervous system (CNS) [1]. A prerequisite for this is an appropriate functional differentiation of immature neural cells into electrophysiologically active neurons. As nearly all CNS diseases involve acute and chronic inflammatory processes [2], it is crucial to understand NSPC development under inflammatory conditions to better realize their full potential. IFNγ is a key inflammatory cytokine, mainly produced by cytotoxic CD8^+ ^T-cells and natural killer cells in the course of neurological diseases like cerebral traumata [3], stroke [4] or multiple sclerosis [5]. Beside the observation that IFNγ-activated microglial cells induce neurogenesis [6], IFNγ has also been reported to exert beneficial, pro-neurogenic effects on NSPC development *in vitro *and *in vivo *in a number of recent publications independently of microglial cells [7-9]. However, a hint that IFNγ might be involved in potentially harmful developmental dysregulation was detected in a number of reports [10-12] and from its tumor-initiating role, since embryonic mice over-expressing IFNγ develop medulloblastomas [13], indicating that IFNγ may also be involved in malignant transformation of neural precursor cells.

In the present study, we demonstrated that IFNγ induces an abnormal immunocytochemical phenotype in NSPCs with simultaneous expression of neuronal and glial markers. Furthermore, IFNγ led to a dysregulated gene expression as well as dysfunctional electrophysiological properties. Additionally, we finally present evidence that IFNγ exposure to NSPCs during an initial 7-day proliferation period dramatically impairs the subsequent development of functional neuronal networks as recorded by the microelectrode array technology. Our data clearly indicate that IFNγ compromises neurogenesis. Thus, its role during inflammatory processes should be reassessed and IFNγ suppression during brain pathology possibly supports functional neurogenesis.

## Results

### IFNγ receptors 1 and 2 are expressed in NSPCs and their differentiated progeny

**Figure 1:**
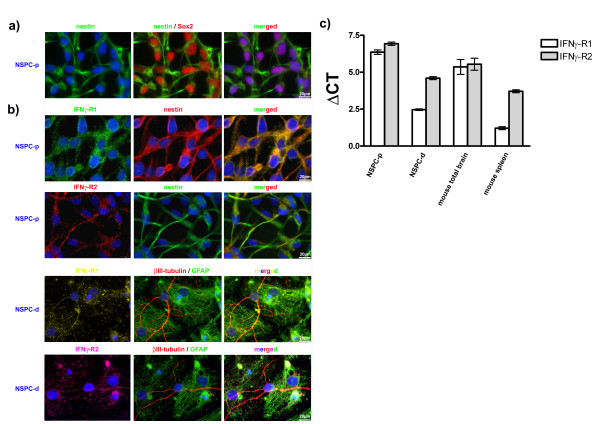
**NSPCs express receptors for IFNγ**. In a: Photomicrographs of proliferating NSPCs (NSPC-p) immunocytochemically labeled against Sox2 and nestin. In b: Photomicrographs of proliferating (NSPC-p) or differentiated (NSPC-d) NSPCs with indicated immunocytochemical markers are given showing that both receptors are expressed on individual cells. In c: real-time quantitative PCR with primers specific for IFNγ-R1 or IFNγ-R2 illustrate the expression of both IFNγ receptors on proliferating (NSPC-p) or differentiated (NSPC-d) NSPCs. Controls are spleen or total brain homogenates. Values are means +/- standard error of mean (SEM). Experiments were performed in triplicate and repeated independently at least three times.

Experiments were performed either with proliferating NSPCs under the influence of growth factors which expressed immature neural markers like Sox2 and nestin (Figure 1A) or with the differentiated progeny of NSPCs that lost their immature markers and instead expressed βIII-tubulin or GFAP (Figure 1B). The signal transduction process of the proinflammatory cytokine IFNγ starts with binding to the IFNγ receptor (IFNGR). This receptor comprises two ligand-binding IFNG-R1 chains which are associated to two signal-transducing IFNG-R2 chains. Both domains of the receptor belong to the class II cytokine receptor family. To study effects of IFNγ on NSPCs and their differentiated progeny, we confirmed the expression of IFNG-R1 and IFNG-R2 in proliferating or differentiated NSPC cultures (Figure 1). We performed immunocytochemical experiments to demonstrate the expression on protein level (Figure 1B). Then we compared the mRNA expression levels of both receptor domains by means of real-time quantitative PCR in various mouse tissues in comparison to proliferative or differentiated NSPCs (Figure 1C). Our results indeed confirmed the presence of both receptor domains in proliferative as well as differentiated NSPCs.

### IFNγ reduces the population extent of NSPCs

**Figure 2:**
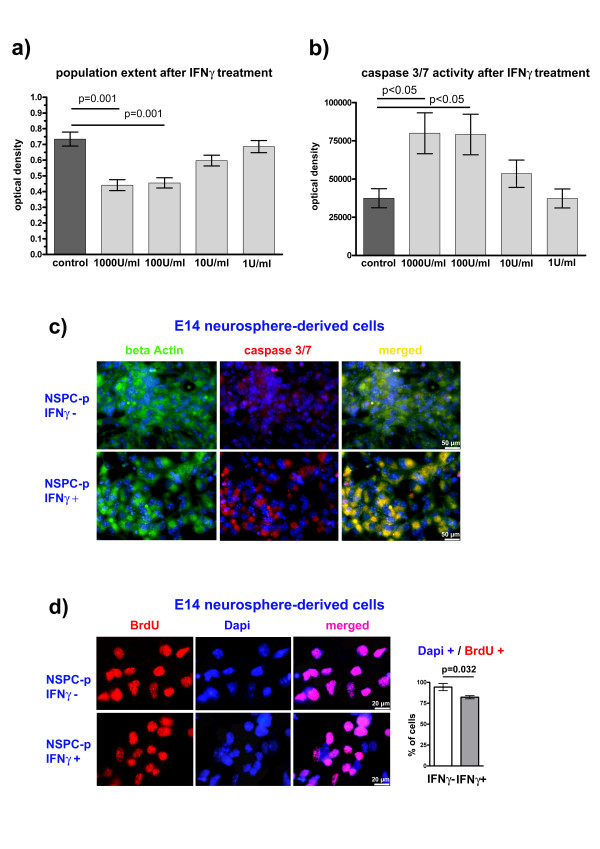
**IFNγ reduces the population extent of NSPCs**. In a: Optical densities [9] correlate to the extent of NSPC populations after a 48-hour IFNγ treatment with indicated concentrations. Values are means +/- standard error of mean (SEM). Experiments were performed in triplicate and repeated independently at least three times. In b: Optical densities correlate to caspase 3/7 activity after a 48-hour IFNγ treatment with indicated concentrations. Values are means +/- standard error of mean (SEM). Experiments were performed in triplicate and repeated independently at least three times. In c: Photomicrographs of caspase 3/7 immunocytochemistries are given for proliferating (NSPC-p) NSPCs with or without IFNγ treatment. Beta Actin labeling visualizes cytoplasmatic structures to better correlate the caspase 3/7 signal to single cells. In d: Photomicrographs of BrdU labelings are given to visualize the amount of proliferating cells with or without IFNγ treatment. Additionally, quantification of BrdU^+ ^cells is given. Values are means +/- standard error of mean (SEM). Experiments were performed in triplicate and repeated independently at least three times. DAPI^+ ^nuclei are given in blue.

To investigate effects of IFNγ on the extent of NSPC populations we performed an MTT-assay. We could demonstrate that 100 or 1000 Units of IFNγ/ml led to significant reductions in the population extent during 48 hours under proliferative conditions (Figure 2A). To verify whether cytotoxic or apoptotic mechanism were involved, we verified caspase 3/7 activity during IFNγ exposure and found a significant increase when caspase activity was measured by means of the Caspase-Glo 3/7 assay (Figure 2B). Also an increased immunocytochemical labeling against caspase 3/7 protein (Figure 2C) suggested an induction of apoptotic pathways in NSPCs after IFNγ treatment. To detect possible anti-proliferative influences of IFNγ on NSPC populations, we performed BrdU labelings. Here, we were able to detect an additional slight but significant anti-proliferative effect of IFNγ (Figure 2D). Together, these data show that IFNγ exerts apoptotic and anti-proliferative effects on NSPCs that together lead to reduced population extents even under the influence of FGF-2.

### IFNγ induces an abnormal phenotype in NSPCs

**Figure 3:**
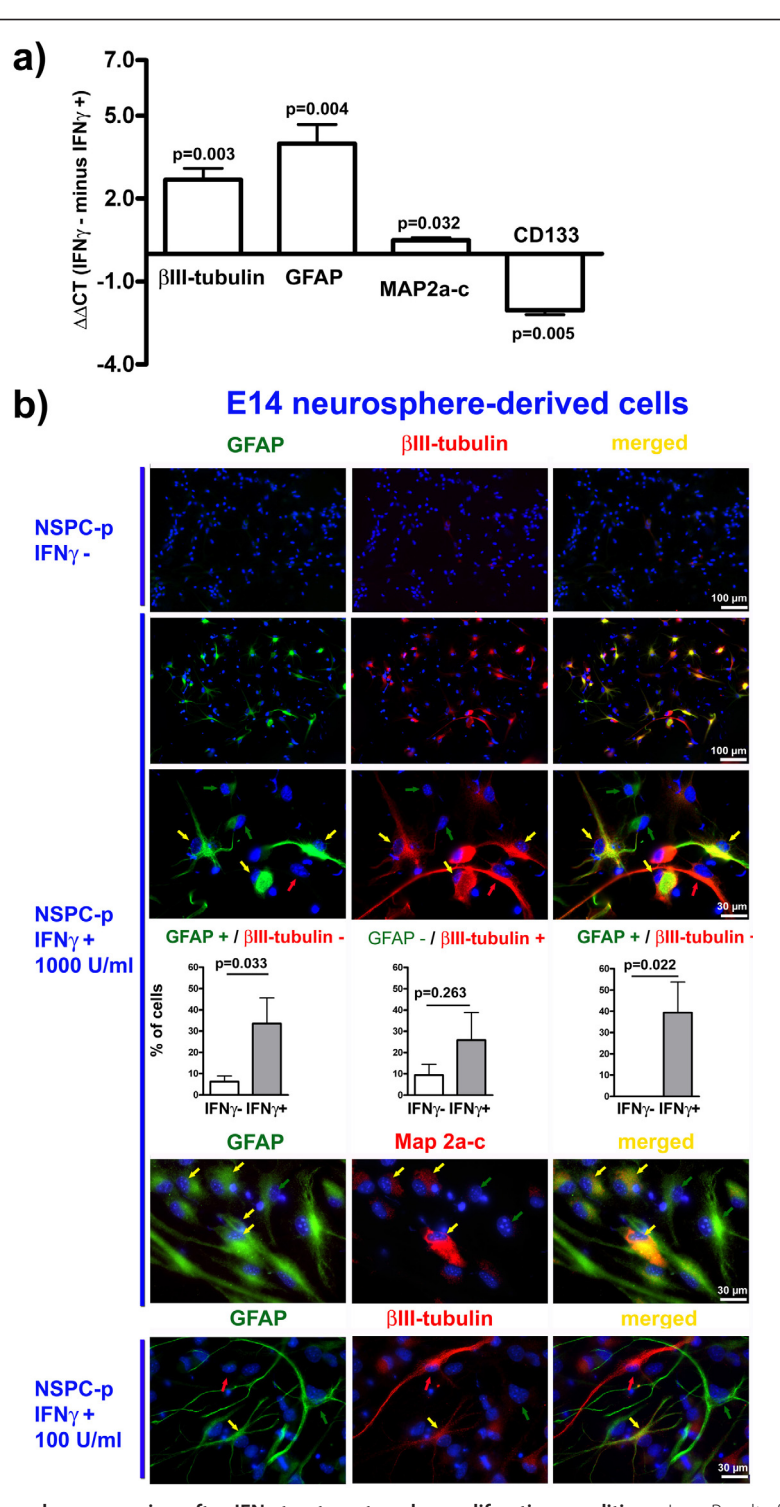
**Cell type-specific marker expression after IFNγ treatment under proliferative conditions**. In a: Results from real-time quantitative PCRs expressed as ΔΔCT (IFNγ- minus IFNγ+) of PBS-treated control and IFNγ-treated groups for the indicated markers of proliferating NSPCs. Higher values indicate a higher gene expression. Values are means +/- standard error of mean (SEM). Experiments were performed in triplicate and repeated independently at least three times. In b: Photomicrographs of proliferating NSPCs (NSPC-p) with indicated immunocytochemical markers (GFAP, βIII-tubulin, Map2a-c). Yellow arrows mark GFAP^+^/βIII-tubulin^+ ^cells, the red arrow marks a GFAP^-^/βIII-tubulin^+ ^neuron and green arrows mark GFAP^+^/βIII-tubulin^- ^astrocytes. DAPI^+ ^nuclei are given in blue. Diagrams show the percentages of immuno-positive cells from all DAPI^+^-cells with (IFNγ+) or without (IFNγ-) IFNγ treatment as indicated. Values are means +/- standard error of mean (SEM). Experiments were performed in triplicate and repeated independently at least three times.

**Figure 4:**
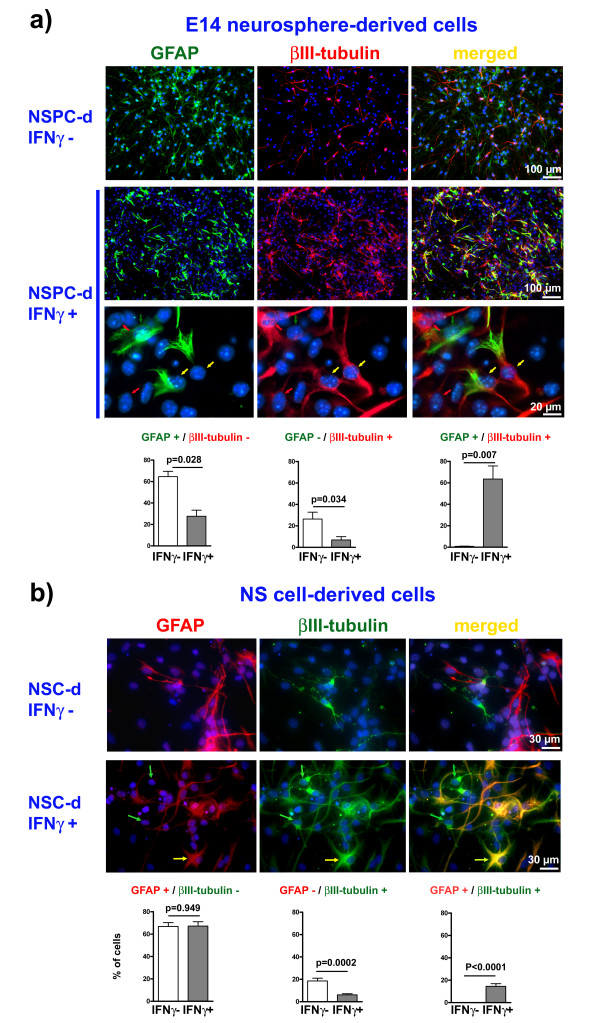
**Cell type-specific marker expression after IFNγ treatment during differentiation**. In a: Photomicrographs of NSPC populations after a differentiation period of 7 days (NSPC-d) are given with indicated immunocytochemical markers and the corresponding quantification of immuno-positive cells from all DAPI^+ ^cells with (IFNγ+) or without (IFNγ-) IFNγ treatment as indicated. Values are means +/- SEM. Experiments were performed in triplicate and repeated independently at least three times. In b: Photomicrographs of mouse embryonic stem cell-derived neural stem cells (NS cells) differentiated for 7 days (NSC-d) with indicated immunocytochemical markers with (IFNγ+) or without (IFNγ-) IFNγ treatment are given. Yellow arrows mark GFAP^+^/βIII-tubulin^+ ^cells. Red arrows mark GFAP^+^/βIII-tubulin^- ^neurons and green arrows mark GFAP^-^/βIII-tubulin^+ ^astrocytes. Note the untypical morphology of GFAP^+^/βIII-tubulin^+ ^cells under IFNγ treatment in comparison to control. Values are means +/- standard error of mean (SEM). Experiments were performed in triplicate and repeated independently at least three times.

In the present study, we found that IFNγ treatment of proliferative murine E14 neurosphere-derived NSPCs caused up-regulation not only of transcripts for βIII-tubulin or microtubule-associated protein 2a-c (MAP2a-c), established markers for post-mitotic neurons, but also for the astrocyte marker glial fibrillary acidic protein (GFAP), challenging the prevalent view of a predominatly pro-neurogenic effect of IFNγ. At the same time, IFNγ executed a down-regulation of CD133, a marker for immature NSPCs, which indicated a robust activation of differentiation programs despite the presence of growth factors (Figure 3A). Immunocytochemical experiments confirmed these results: on the one hand, we could detect an anti-proliferative effect of IFNγ as numbers of BrdU^+ ^cells decreased (Figure 2D). On the other hand, we detected a robust increase in the number of cells expressing neuronal and glial specific proteins after IFNγ treatment (Figure 3B). Surprisingly, in addition to GFAP^-^/βIII-tubulin^+ ^or GFAP^+^/βIII-tubulin^- ^cells, a considerable number of NSPCs (39.3 ± 14.5% of all cells) co-expressed GFAP and βIII-tubulin after a 3-day treatment with 1000 U/ml IFNγ (Figure 3B). GFAP^+^/βIII-tubulin^+ ^cells in comparable numbers were also detectable after IFNγ treatment with only 100 U/ml (Figure 3B). Using a concentration of 100 U/ml IFNγ led to a slightly weaker induction of GFAP and βIII-tubulin immunoreactivity in individual cells while numbers of cells showing at all this phenomenon were similar with 100 or 1000 U/ml. This phenomenon was absolutely rare (< 0.01%) in the absence of IFNγ (Figure 3B). In addition, IFNγ treatment induced simultaneous expression of GFAP and the post-mitotic neuronal markers MAP2a-c in a large percentage of cells (GFAP^+^/MAP2a-c^+^: 73.6 ± 5.7%), illustrating that the co-expression of glial and neuronal markers is not restricted to βIII-tubulin. We next investigated the influence of IFNγ treatment during a 7-day differentiation period after growth factor withdrawal from NSPC cultures to elucidate effects on cell maturation. Again, we detected cells co-expressing GFAP and βIII-tubulin (Figure 4A). Moreover, numbers of GFAP^-^/βIII-tubulin^+ ^or GFAP^+^/βIII-tubulin^- ^cells in IFNγ treated cultures differentiated for 7 days were significantly lower than without IFNγ Figure 4A), which is in direct contrast to the expected pro-neurogenic role of IFNγ. As terminal neuronal differentiation could take longer than 7 days, we also cultured NSPC populations without growth factors for 14 or 21 days under the influence of IFNγ. Also here we found GFAP^+^/βIII-tubulin^+ ^cells indicating that this phenotype is stable during 14 or 21 days under differentiation conditions (data not shown). To verify if this phenomenon depends on more restricted precursors being present in neurosphere-derived populations, we generated homogenous cultures of multipotent neural stem cells (NS cells). These populations were generated from murine embryonic stem cells. Also in NS cell cultures, GFAP^+^/βIII-tubulin^+ ^cells were induced even though the part of NS cell-derived GFAP^+^/βIII-tubulin^+ ^cells was smaller in comparison to neurosphere-derived GFAP^+^/βIII-tubulin^+ ^cells. This shows that IFNγ can generate this abnormal phenotype from immature neural stem cells independently of the presence of neuronal or glial precursors (Figure 4B). Thus, our experiments suggest that IFNγ can drive the differentiation of NS cells or NSPC populations towards an immunocytochemically abnormal marker profile, indicating a genetic and/or functional dysregulation.

### IFNγ induces an abnormal down-stream signaling in NSPCs

**Figure 5:**
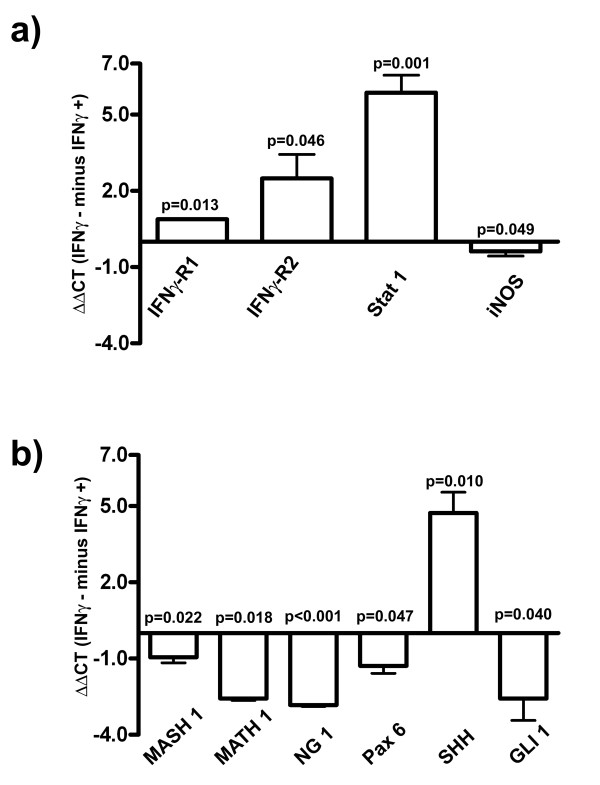
**Down-stream signaling analysis after IFNγ treatment under proliferative conditions**. In a and b: Results from real-time quantitative PCRs expressed as ΔΔCT (IFNγ- minus IFNγ+) of PBS-treated control and IFNγ-treated groups for the indicated markers of proliferative NSPCs. Higher values indicate a higher gene expression. Values are means +/- standard error of mean (SEM). Experiments were performed in triplicate and repeated independently at least three times.

To study changes in IFNγ receptor expression, we performed quantitative real-time PCR after a 3-day IFNγ treatment of proliferative NSPCs. We detected increases in the transcript numbers of IFNγ-receptor1 and IFNγ-receptor2 (Figure 5A), as well as the IFNγ-related down-stream factor signal transducers and activators of transcription 1 (Stat1). However, inducible nitric oxide synthase (iNOS), a gene product, which is usually up-regulated as a result of IFNγ signaling, was down-regulated, revealing another surprising effect of IFNγ on NSPCs (Figure 5A). We investigated IFNγ-induced changes of pro-neural basic helix-loop-helix (bHLH) genes and the neurogenic transcription factor Pax6, which are important for neuronal determination. Supporting the notion that IFNγ does not promote neuronal determination, we found that Math1, Mash1, Neurogenin1 and Pax6 were down-regulated in NSPCs after IFNγ treatment (Figure 5B). Further, while IFNγ treatment significantly up-regulated SHH in NSPCs, Gli1 was down-regulated, an effect that was unanticipated (Figure 5B).

### GFAP^+^/βIII-tubulin^+ ^cells exhibit non-neuronal and non-astrocytic functional properties

**Figure 6:**
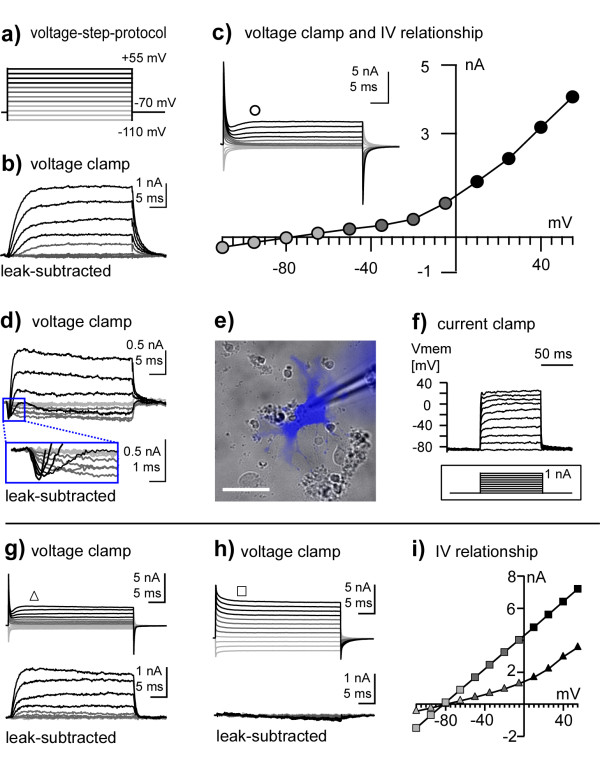
**Patch-clamp results of IFNγ treated NSPCs**. In a: Voltage-step protocol used to probe cells for voltage-activated conductances (see current traces in panels b-d, g, and h). In b, c: GFAP^+^/βIII-tubulin^+ ^cells typically exhibited an outward rectifying current voltage (IV) relationship (13/14 cells), IV-relationship was determined from non-leak-subtracted traces 10 ms after the voltage step (circle). In d: In 4/14 GFAP^+^/βIII-tubulin^+ ^cells, voltage-gated inward currents were observed in addition to outward currents. In e: Image of cell filled with Alexa Fluor350 and later identified as GFAP^+^/βIII-tubulin^+ ^(scale bar 30 μm, recordings illustrated in figure d) and f)). In f: None of the 14 GFAP^+^/βIII-tubulin^+ ^cells exhibited action-potential-like events, upon depolarization to at least -30 mV evoked by current injections (inset). In g, h: Membrane currents typically observed in GFAP^+^/βIII-tubulin^- ^cells (each recorded in 3/6 cells). In i: IV-relationships taken from non-leak-subtracted traces illustrated in g) (triangle) and h) (square).

To elucidate if the unusual effects of IFNγ treatment on the differentiation of NSPCs are accompanied by an atypical functional phenotype, we next analyzed basic electrophysiological properties of GFAP^+^/βIII-tubulin^+ ^cells. For this purpose we combined whole-cell patch-clamp with subsequent immunocytochemistry to unambiguously identify recorded cells. GFAP^+^/βIII-tubulin^+ ^cells in IFNγ-treated proliferative (n = 5) or differentiated (n = 9) cultures almost exclusively exhibited an outward rectifying current-voltage (IV) relationship (13/14 cells, Figure 6a-c). Small inward currents were observed in 4/14 cells (Figure 6d). When challenging GFAP^+^/βIII-tubulin^+ ^cells within differentiated cultures with current injections in current-clamp mode, none of these cells exhibited action potentials when depolarized to up to a membrane potential of approximately +30 mV (n = 7, Figure 6e, f). GFAP^+^/βIII-tubulin^- ^cells in differentiated cultures either exhibited an outward rectifying IV relationship, similar to GFAP^+^/βIII-tubulin^+ ^cells (3/6; Figure 6g, h, i), or a linear IV-relationship that completely lacked voltage-dependent conductances (n = 3/6; Figure 6g, h, i). Such I/V relationships are typical for mature classical astrocytes [14] and were never observed in GFAP^+^/βIII-tubulin^+ ^cells. Our electrophysiological results, thus, demonstrate that GFAP^+^/βIII-tubulin^+ ^cells are functionally distinct from mature astrocytes as well as neurons.

### IFNγ treatment impaired the formation of in vitro-functional neural networks

**Figure 7:**
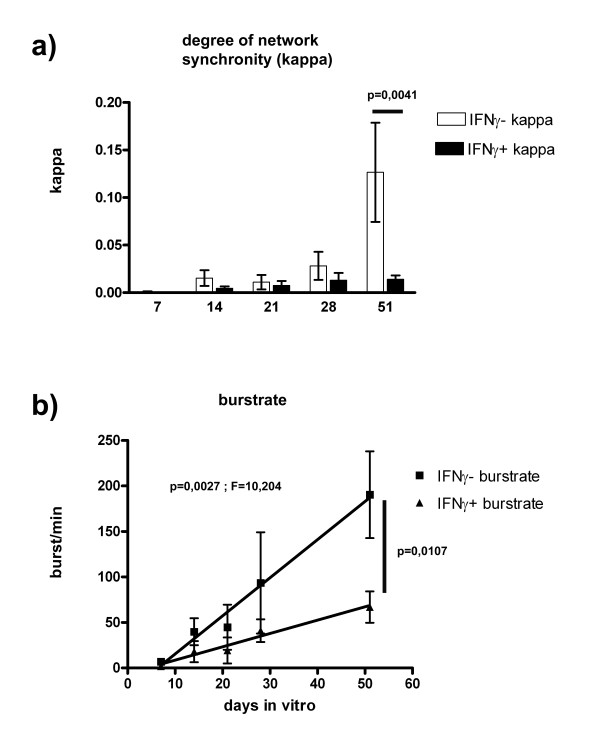
**MEA-recordings of mouse ES cell-derived neural populations**. In a + b: Different electrophysiological parameters of MEA-recordings with or without IFNγ treatment in differentiated mouse ES cell-derived neural precursor cells at indicated time points are illustrated. In a: The synchrony of the neural network which is expressed in kappa values. In b: Bursts per minute are given. In a, the student´s t-test is applied. In b, linear regression is given with p- and F-values and, additionally, the t-test is performed for the 51 days in vitro time point. Values are means +/- standard error of mean (SEM). Experiments were performed in triplicate and repeated independently at least three times.

To ultimately verify the effect of IFNγ on NSPC populations, we performed experiments using the microelectrode array (MEA) technology, that is able to detect functional neuronal network activity of entire neural populations. Immature ES cell-derived nSFEB aggregates consisting of a mixture of immature neural precursor cells were exposed to IFNγ during their initial 7-day proliferation period under the influence of FGF-2. Hereafter, IFNγ was removed and differentiation was initiated by FGF-2 withdrawal. By this paradigm, IFNγ treatment selectively hit developmental processes of ES cell-derived NSPCs, while subsequent synapse formation and other maturational processes were excluded from direct IFNγ influences. After the initial 7-day period, maturation of cultures was observed for additional 44 days. Normally, during the 3^rd ^and 4^th ^week, cultures start to develop functional neuronal networks that show increasing burst activity that finally ends in oscillating and synchronous neuronal network activity (Figure 7, IFNγ-). This synchrony of action potential bursts in spatially distributed neurons is expressed by the kappa value, with increasing values showing higher network synchrony. By this experiment we could show that a 7-day exposure to IFNγ during a developmentally sensitive period of immature NSPCs under the influence of FGF-2 sustainably impairs the subsequent generation of functional neuronal networks as burstrate and kappa levels were significantly smaller in comparison to untreated populations (Figure 7). As neuronal network formation can also be impaired by low cell densities, we verified this factor under IFNγ-treated conditions. However, even though IFNγ led to a reduced population extent, the 7-day proliferation period was by far sufficient to allow for the growth of morphologically dense and confluent neural cell populations.

## Discussion

Our results shed new light on the effects of IFNγ on NSPCs. Until now, IFNγ-related up-regulation of βIII-tubulin was interpreted as a beneficial enhancement of neurogenesis [7-9]. The present study disproves this view and shows that IFNγ instead promotes an abnormal NSPC-derived cellular phenotype that does not relate to classical neurons or astrocytes and that appears to be dysregulated in terms of functional and molecular properties. IFNγ treatment leads to the expression of both, class III βtubulin and GFAP in ~40% of NSPC which is abnormal and, even after differentiation, not linked to mature neuronal or astrocytic electrophysiological function. Class III βtubulin isotype is usually considered specific for post-mitotic neurons, and such aberrant expression has so far only been noted in gliomas [15,16] or dysregulated tumorigenic neural stem cells [17,18]. Walton and colleagues even report some unusual co-expression of βIII-tubulin and GFAP in tumorigenic neural stem cells, a phenomenon similar to that detected here after IFNγ treatment of regular NSPCs. The aspect of IFNγ-mediated NSPC dysregulation is further substantiated in the present report by an up-regulation of SHH which is paralleled by down-regulation of Gli1 which has been reported to be consistently up-regulated in the course of SHH signaling [19]. As expression patterns of neurogenic niche morphogenes like SHH or Gli1 are generally tightly regulated during CNS development, its disturbance points to misguided development or again tumorigenesis [13,19]. Thus, for the first time, we directly illustrated a possible link between IFNγ, NSPCs and cellular abnormalities similar to that observed in tumor cells strongly supporting the view that inflammation might be involved in tumor generation via neural stem cells. Additionally, the IFNγ-related down-regulation of iNOS in NSPC cultures is untypical as it is known that IFNγ normally induces iNOS [20]. Our electrophysiological findings illustrate the importance of an additional functional control of morphological/immunocytochemical observations as the up-regulation of βIII-tubulin in differentiated NSPC-derived cells, which was interpreted as enhanced neurogenesis in different studies [7,9], was not paralleled by neuronal electrophysiological behavior. Further, the increase in GFAP^-^/βIII-tubulin^+ ^neurons after IFNγ treatment of proliferating cultures was not significant in the present study and after differentiation under the impact of IFNγ we even found significantly less GFAP^-^/βIII-tubulin^+ ^neurons. Interestingly, a similar observation was described previously [21]. We found that those βIII-tubulin expressing cells that significantly increased in numbers after IFNγ treatment of proliferating or differentiating cultures were also GFAP positive and exhibited electrophysiological properties that were neither typical for mature astrocytes nor for neurons. We demonstrated this by careful correlating electrophysiological data of patched cells with their immunocytochemical phenotype. These molecular and functional IFNγ effects on NSPCs indicate a profoundly compromised cell function or, alternatively, a new IFNγ-induced NSPC-derived neural cell of unknown function. Interestingly, ectopic expression of IFNγ during early stages of CNS development induces medulloblastomas via SHH overexpression [13] pointing towards a general dysregulating effect of IFNγ on NSPCs during development or disease.

To investigate functional neural development under controlled conditions, with and without IFNγ-treatment, we electrophysiologically measured the development of functional neuronal networks starting from ES cell-derived immature neural precursor cell cultures. Usually, network activity progressively develops over time as a result of a complex interaction of a multitude of factors that converge to an integrated functional entity [22]. It depends on efficient synapse formation and function of an entire neuronal population. If using immature neural precursor populations as developmental starting point, basic aspects of functional neural development can be measured. In contrast, mature ES cell-derived functional neuronal networks can be used to detect acute functional consequences due to changes in extracellular composition. These investigations then affect already active neuronal networks. For instance, they showed to reversibly alter their network function under the influence of different cerebrospinal fluid specimens [23]. We chose a paradigm in which the influence of IFNγ selectively affected the initial proliferation period of cultures that were subsequently held under normal differentiating conditions. IFNγ-treated cultures showed a significantly impaired development of neuronal network function, impressively pointing to an IFNγ-related, profoundly altered functional development of neural precursor populations.

## Conclusion

Thus, we speculate that abnormally high IFNγ production during development and CNS diseases might impair functional neuronal development in fetal neurogenesis or adult regeneration and propose to inhibit IFNγ effects on NSPCs as a means to effectively support their developmental and regenerative potential.

## Materials and methods

### Neurosphere cultures

Neurospheres were generated from fourteen-day-old wild-type C57BL/6J mouse embryos. Ganglionic eminences were removed, mechanically dissociated and seeded in DMEM/F12 culture medium (1:1; Invitrogen, Karlsruhe, Germany) containing 0.6% Glucose (Sigma-Aldrich, Hamburg, Germany), glutamine (2 mM; Invitrogen), sodium bicarbonate (3 mM; Invitrogen), Hepes buffer (5 mM; Invitrogen) and B27 (20 μl per ml; Invitrogen). For generation and expansion of neurosphere cells, epidermal growth factor (EGF) (Tebu-bio, Le Perray en Yvelines Cedex, France) and basic fibroblast growth factor-2 (FGF-2) (Tebu-bio) were added to a final concentration of 20 ng per ml each.

### Generation of embryonic stem cell-derived neural stem cells

Undifferentiated ES cells (SV-129, ATCC, Millipore, Billerica, USA) were grown under feeder-deprived conditions in the presence of 1000 U/ml leukemia inhibitory factor (LIF, Millipore) and 20% fetal bovine serum (FBS, HyClone, Thermo Fisher Scientific, Schwerte, Germany) in ES cell medium described elsewhere [24]. Neural differentiation of immature ES cells into neural stem cell (NS cells) was performed according to modified protocols [22,25].

### IFNγ treatment and immunocytochemistry

For immunocytochemistry, neurosphere cells or ES cell-derived NS cells were dissociated to a single cell suspension and plated on poly-L-ornithine (PLO; 0.001%; Sigma-Aldrich) and fibronectin (5 μg/ml; Tebu-bio) coated cover slips (VWR International, Darmstadt, Germany) at a density of 50 × 10^3 ^cells per ml. After 3 days under the influence of EGF and FGF-2 (20 ng/ml both Tebu-bio), cells were assigned to the different experimental groups. To verify the marker expression of undifferentiated (proliferating) neural populations under control or IFNγ treated conditions, cultures were kept under the influence of EGF/FGF-2 without or with IFNγ (1000 U/ml; Millipore) until fixation after further 3 days (NSPC-p -IFNγ/+IFNγ). To verify cell-type specific marker expression in differentiated cultures, growth factors were withdrawn and then, cells were treated for 7 days without or with IFNγ until fixation (NSPC-d -IFNγ/+IFNγ). For control experiments, only phosphate-buffered saline solution (PBS; 1X; Invitrogen) was added to the medium. Primary antibodies used at 4°C overnight were monoclonal mouse antibodies to 5-bromo-2-deoxyuridine (BrdU; 1:1000, Sigma-Aldrich), βIII-tubulin (Tuj1; 1:500; R&D Systems, Minneapolis, USA or 1:800, Abcam, Cambridge, UK), Map2a-c (1:2000; Sigma-Aldrich), Sox2 (1:50; R&D Systems), IFNγ-R1 (1:500; Santa Cruz Biotechnology) and beta Actin (1:100; Millipore) and polyclonal rabbit antibodies to glial fibrillaric acid protein (GFAP) (1:500; Dako, Hamburg, Germany or 1:1000; Abcam), caspase (1:100; Cell Signaling), IFNγ-R2 (1:500; Santa Cruz Biotechnology) and nestin (1:200; Covance). BrdU labeling is described elsewhere (Wellen et al., 2009). For detection of primary antibodies, fluoresceine-isothiocyanate (FITC; 1:500; Millipore) and indocarbocyanine (Cy3; 1:800; or Cy5; 1:200; Millipore) coupled secondary antibodies were used. For negative controls, primary antibodies were omitted in each experiment. To measure the total population of cells, Dapi positive cell nuclei were counted. On every cover slip, at least 100 cells were counted.

### MTT-Assay

To analyze the population extent of NSPCs, the optical density [9], indicative of conversion of 3-(4, 5-dimethylthiazol-2-yl)-2, 5-diphenyltetrazolium bromide (MTT; Sigma-Aldrich) into formazan crystals which takes place in live cells only, was determined after IFNγ treatment at decreasing concentrations as indicated. An OD value of 0.5 represents approximately 50,000, and an OD value of 1.0 represents approximately 100,000 live NSPCs. The population extent was measured after 48 hours of IFNγ treatment as indicated.

### Caspase-activity Assay

For detection of caspase 3/7 activity after IFNγ treatment, we used the Caspase-Glo 3/7 assay (Promega, Madison, USA). Proliferating cultures were treated with decreasing concentrations of IFNγ as indicated. Adding the assay components to cultivated cells leads to cell lysis and release of caspase 3/7. Caspase 3/7 is capable of cleaving a tetrapeptide sequence substrate; this is dismantled by luciferase which is a component of the assay. The resulting light emission is then a measure of caspase activity.

### Quantitative real-time PCR

RNeasy Kit (Qiagen) was used for RNA isolation of cultured NSPCs. Then a reverse transcription into cDNA (ABI, Darmstadt, Germany) was performed. Quantitative real-time PCR was carried out by the usage of the 7500 fast or 7500 quantitative real-time PCR cycler (ABI, Darmstadt, Germany). Either SYBR green master mix (Qiagen) or equivalent chemistry from another supplier (Quantace, London, UK) was used. The specific primer for genes of interest or the housekeeping gene (glyceraldehyde-3-phosphate dehydrogenase, GAPDH) was either purchased (QuantiTect primer assays, Qiagen) or self designed (BioTEZ, Berlin, Germany). The genes of interest (target gene) in IFNγ-treated groups or control groups (PBS-treated) were analyzed in at least 3 independent cultures in triplicate each. Every experiment in IFNγ-treated or control (PBS-treated) groups provided delta CT values (ΔCT: gene of interest minus housekeeping gene). The presented graphs are ΔΔCT values:

$$\Delta \Delta \text{CT}={\left({\text{Ct}}_{\text{target gene}}–{\text{ Ct}}_{\text{housekeeping gene}}\right)}_{\text{PBS-treated}}-{\left({\text{Ct}}_{\text{target gene}}-{\text{ Ct}}_{\text{housekeeping gene}}\right)}_{\text{IFN}\gamma \text{-treated}}$$

### Patch-clamp recordings

Somatic whole-cell patch-clamp recordings were carried out using an Axopatch 200B amplifier (Molecular Devices, Sunnyvale, CA, USA) coupled to a personal computer via a digidata 1322A interface (Molecular Devices). Data were acquired at 10 kHz using PClamp 8.2 software (Molecular Devices). Patch pipettes were pulled from borosilicate glass (Hilgenberg, Waldkappel, Germany) and had a resistance of 3-6 MΩ when filled with intracellular solution containing (in mM): 120 K-MeSO_3_, 32 KCl, 10 HEPES (N-(2-Hydroxyethyl)piperazine-N'-(2-ethanesulfonic acid), 4 NaCl, 4 Mg-ATP and 0.4 Na-GTP, 1 Alexa Fluor 350 (Molecular Probes/Invitrogen), pH 7.30 (calculated liquid junction potential: 12.5 mV). Cells were held at membrane potentials of -70 mV. To separate passive conductances from voltage-gated currents, online leak subtraction (P/4) was performed. Extracellular solution during patch-clamp experiments contained in mM: 125 NaCl, 2.5 KCl, 2 CaCl_2_, 1 MgCl_2_, 1.25 NaH_2_PO_4_, 26 NaHCO_3_, and 20 glucose, bubbled with 95% O_2 _and 5% CO_2 _to result in a pH of 7.4. Patch-clamp data were processed and analyzed using IGOR Pro-Software (WaveMetrics, Inc., Lake Oswego, OR). After the recordings, patch-pipettes were carefully withdrawn and coverslips were transferred into 4% paraformaldehyde for 20 minutes at room temperature. Thereafter coverslips were kept in PBS (Invitrogen) at 4 °C until they were processed for GFAP and βtubulin immunocytochemistry. By means of fluorescence at 350 nm electrophysiologically recorded cells were identified and assigned either to GFAP^+^/βIII-tubulin^+ ^or GFAP^+^/βIII-tubulin^- ^cells.

### Microelectrode array recordings

For microelectrode array (MEA) recordings, 5 to 10 neural precursor cell-enriched, serum-free, floating embryoid body-like aggregates (nSFEBs) [22] were seeded on poly-D-lysine (PDL, 15 μg/ml, Sigma-Aldrich, Germany) and laminin (15 μg/ml, Sigma-Aldrich, Germany) coated MEAs with a square grid of 60 planar Ti/TiN electrodes (30-μm diameter, 200-μm spacing) and an input impedance of <50 kÙ according to the specifications of the manufacturer (Multi Channel Systems, Reutlingen, Germany). Signals from all 60 electrodes were simultaneously sampled at 25 kHz, visualized and stored using the standard software MC Rack provided by Multi Channel Systems. Spike and burst detection was performed off-line by custom-built software (Result, Düsseldorf, Germany). nSFEBs were kept for 7 days after plating under the influence of FGF-2 (20 ng/ml, PeproTech) only (IFN-γ - group) or together with IFN-γ (1000 U/ml; IFN-γ + group). After 7 days, FGF-2 and IFN-γ were removed from the medium to induce terminal differentiation. For long-term culture, ES cell-derived neuronal networks were kept in DMEM/F12 (Gibco) supplemented with N2, B27 and Glutamax (all Invitrogen). MEA recordings were performed at the indicated time points.

### Statistical analyses

Experiments were repeated with independent cultures at least three times in triplicate each. The resulting data sets were statistically analyzed und illustrated using the GraphPad Prism 4 (GraphPad Software Inc., San Diego, CA, USA, 2003) software. For approval of statistical significance between groups a two-tailed t-test was performed. *P *values < 0.05 were considered to indicate significant differences. For comparison of functional neuronal network development, slopes of linear regressions were calculated with GraphPad Prism 4 and p- and F-values were given.

## Competing interests

The authors declare that they have no competing interests.

## Authors' contributions to the manuscript

MD and JW conceived and designed the manuscript. MD supported the study financially. HPH supported the study administratively. JW, SDH, SI and JMW collected and assembled the data. JW and MD wrote the manuscript. The manuscript was finally approved by MD, HPH and CRR. CRR conceived and afforded the patch clamp recordings. SDH performed and analysed the patch clamp recordings. SI performed the MEA recordings.

All authors read and approved the final manuscript.
